# Thapsigargin—From *Thapsia* L. to Mipsagargin

**DOI:** 10.3390/molecules20046113

**Published:** 2015-04-08

**Authors:** Trine Bundgaard Andersen, Carmen Quiñonero López, Tom Manczak, Karen Martinez, Henrik Toft Simonsen

**Affiliations:** Department of Plant and Environmental Sciences, Faculty of Science, University of Copenhagen, Thorvaldsensvej 40, 1871 Frederiksberg, Denmark; E-Mails: trba@plen.ku.dk (T.B.A.); c.quinonero@plen.ku.dk (C.Q.L.); tmanczak@gmail.com (T.M.); kama@plen.ku.dk (K.M.)

**Keywords:** thapsigargin, mipsagargin, *Thapsia garganica*, pharmacology, biosynthesis, traditional use, sesquiterpene lactone

## Abstract

The sesquiterpene lactone thapsigargin is found in the plant *Thapsia garganica* L., and is one of the major constituents of the roots and fruits of this Mediterranean species. In 1978, the first pharmacological effects of thapsigargin were established and the full structure was elucidated in 1985. Shortly after, the overall mechanism of the Sarco-endoplasmic reticulum Ca^2+^-ATPase (SERCA) inhibition that leads to apoptosis was discovered. Thapsigargin has a potent antagonistic effect on the SERCA and is widely used to study Ca^2+^-signaling. The effect on SERCA has also been utilized in the treatment of solid tumors. A prodrug has been designed to target the blood vessels of cancer cells; the death of these blood vessels then leads to tumor necrosis. The first clinical trials of this drug were initiated in 2008, and the potent drug is expected to enter the market in the near future under the generic name Mipsagargin (G-202). This review will describe the discovery of the new drug, the on-going elucidation of the biosynthesis of thapsigargin in the plant and attempts to supply the global market with a novel potent anti-cancer drug.

## 1. The Genus *Thapsia*

### 1.1. Traditional Use and Description

*Thapsia* L. species, otherwise known as deadly carrots, have been used in traditional medicine in the Mediterranean region for thousands of years. Hippocrates and Theophrastus first described the skin-irritating effects and medicinal uses of *Thapsia garganica* L. around 300–400 BC. In 1597, the effect of *Thapsia* was vividly described as “*if a man do stand where the wind doth blow from the plant, the air doth exulcerate and blister the face, and every other bare or naked place that may be subject to his venomous blast and poisonous quality*” [1]. Although this description is an exaggeration, direct skin contact with the plant can result in dermatitis in the form of erythema, small blisters and itching. The resin from the roots and stems of *T. garganica* has been used as a remedy against a number of diseases and maladies: female sterility, pulmonary diseases, catarrh, fever, pneumonia and as a counter irritant for the relief of rheumatic pains [1,2,3]. In 1857 the use of *T. garganica* in Europe was recommended for the treatment of lung diseases, catarrh and rheumatic pains, through the application of a medicinal plaster containing the root resin [4]. In present day Morocco *Thapsia* spp. are still in use in traditional medicine [3,5]. Furthermore, *T. garganica* has been featured in several pharmacopoeias [6].

The effects of *Thapsia* spp. are due to the presence of specialized metabolites, such as sesquiterpenoids, which are found in all members of the genus [7]. In particular, the bioactivity of the sesquiterpenoid thapsigargin extracted from *T. garganica* has been thoroughly investigated. In 1978, for instance, it was shown that thapsigargin functions as a potent histamine liberator when tested on rat mast cells [8]. In addition, the treatment of mammalian cells with thapsigargin was shown to result in raised calcium levels in the cytoplasm and in 1990 thapsigargin was established as an inhibitor of the sarco-endoplasmic reticulum Ca^2+^-ATPase (SERCA) [9]. 

### 1.2. Phylogeny of Thapsia L.

**Figure 1:**
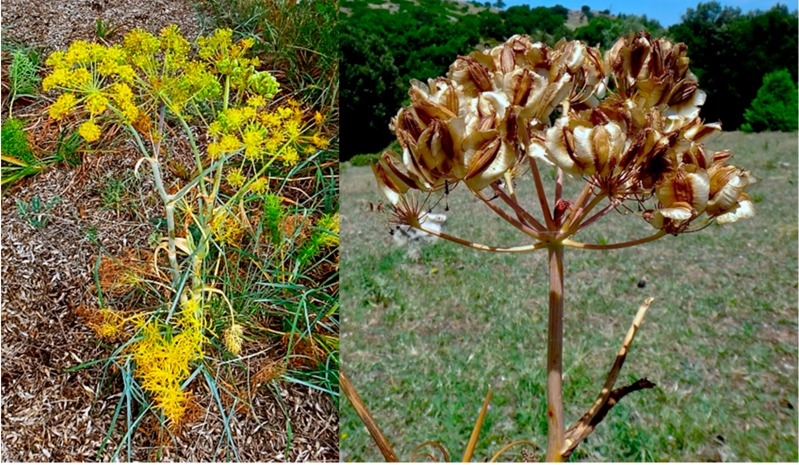
Show the yellow inflorescence of *Thapsia garganica*, and the mature infructescence of the species. Photos by Karen Martinez.

*Thapsia* is a small genus of herbaceous perennials in the Apiaceae family that is widely distributed across the Mediterranean from Portugal and Morocco to Greece and Turkey. The most extensively distributed species within the genus is *T. garganica* [10,11]. *Thapsia* species have characteristic bright yellow flowers, four-winged seeds, large umbels and stems that can reach a height of up to two meters (Figure 1). The genus is currently poorly defined, with frequent errors in species identification or conflicting views on which names define which members of the group. This is especially problematic in the case of users of traditional medicine, where the selection of the wrong plant can lead to the intoxication of patients. In Morocco, for example, where women use the root of what should be *Thapsia villosa* L. in preparations against sterility or to gain weight, cases of vomiting and violent diarrhea have been reported [2], likely due to the use of morphologically similar species. Based on the most recent phylogenetic analysis of *Thapsia*, the genus includes 14 species [10]. Chemotaxonomic revisions, however, are still ongoing and frequently reveal new intra- or interspecies relationships [12]. 

### 1.3. Sesquiterpenoids in Thapsia

Sesquiterpenoids are widespread within the genus *Thapsia*, however, it is thapsigargin and the group of similar compounds, guaianolides, which are of most interest (Table 1). Due to the toxicity of thapsigargin towards mammals, one *in planta* function is expected to be protection of the plants from herbivory. Thapsigargin has only been reported in *T. garganica* [13] and *T. gymnesica* Rosselló and Pujadas [7]. The concentration of thapsigargin varies even within species, seemingly dependent of its locality [13]. Wild plants of *T. garganica* have a concentration of thapsigargin of 0.2%–1.2% of the dry weight of the roots and 0.7%–1.5% of the dry weight of the ripe fruits, whilst the dried stems and leaves contain a total concentration of 0.1%–0.5% and 0.1% respectively [13]. 

**Table 1:** Thapsigargin and similar guaianolides reported within *Thapsia*. The table is amended from Drew *et al.* [14] The top sketch illustrates the core structure of thapsigargin. 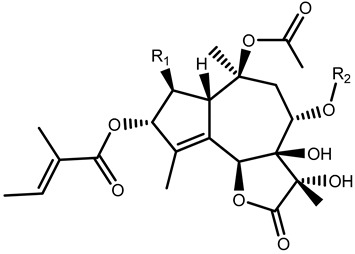

Species		Compound				R1	R2	Reference
***T. garganica* L.**		Thapsigargin				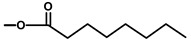	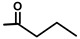	[8]
		Thapsigargicin				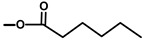	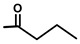	[8]
		Thapsivillosin C-E				See below		[15]
		Thapsivillosin I				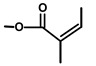	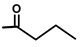	[13]
		Thapsivillosin J				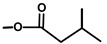	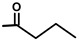	[13]
		Thapsivillosin L				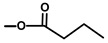	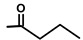	[16]
		Nortrilobolid				H	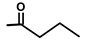	[17]
		Trilobolid				H	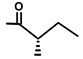	[13,17]
		*not named*				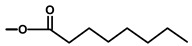	H	[18]
		*not named*				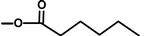	H	[18]
***T. gymnesica* Rosselló & A. Pujadas**		Thapsigargin				See above		[7]
		Thapsigargicin				See above		[7]
		Nortrilobolid				See above		[7]
***T. villosa* L.**		Thapsivillosin A				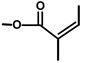	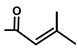	[19]
		Thapsivillosin B				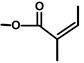	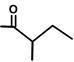	[13]
		Thapsivillosin C				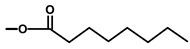	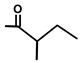	[13]
		Thapsivillosin D				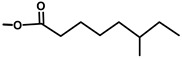	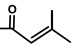	[13]
		Thapsivillosin E				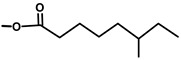	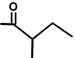	[13]
		Thapsivillosin F				H	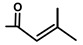	[13]
		Thapsivillosin G				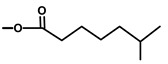	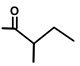	[13]
		Thapsivillosin H				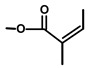 or 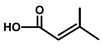 Exact positions undefined		[13]
***T. villosa* L.**		Thapsivillosin K				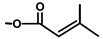	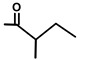	[13]
		Thapsitranstagin				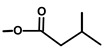	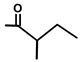	[13,20]
		Trilobolide				See above		[19]
***T. transtagana* Brot.**		Thapsitranstagin				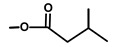	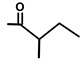	[13,21]
		Thapsivillosin B				See above		[13,21]
		Trilobolid				See above		[13,21]
		Thapsivillosin K				See above		[13,21]
***T. smittii* Simonsen, Rønsted, Weitzel and Spalik**		Thapsivillosin A, B, H				See above		[19]

## 2. Thapsigargin and Guaianolide Biosynthesis in Thapsia

Terpenoids are the largest class of small natural products and are structurally highly diverse, containing metabolites of both general and specialized metabolism [22]. Within the terpenoids, sesquiterpenoids (C_15_) are a large group of specialized metabolites. Thapsigargin is a sesquiterpene lactone and belongs to the subgroup guaianolides. This group has a guaiene backbone and a lactone ring (see Table 1).

### 2.1. The Biosynthesis of Sesquiterpene Lactones

Sesquiterpenoids are built from isopentenyl diphosphate (IPP) and dimethylally diphosphate (DMAPP), which are small molecules consisting of five carbon atoms. IPP and DMAPP, which are used for sesquiterpenoid and sterol biosynthesis, are biosynthesized by the mevalonate (MVA) pathway located in the cytosol. Similar to many other anabolic pathways, acetyl-CoA is used as the starting material and NADPH as an energy source [22]. The second pathway for biosynthesis of IPP and DMAPP is the methylerythritol phosphate (MEP) pathway found in the plastids [23,24]. The MEP pathway generates precursors for carotenoids, chlorophylls, monoterpenoids, and diterpenoids [25,26]. Cross talk has been observed between the two pathways, and studies with labeled precursors in *Daucus carota* L., have revealed the presence of a unidirectional proton symport of IPP from plastids to the cytoplasm in several higher plants [26,27,28,29]. 

In the cytosol, IPP and DMAPP are fused to synthesize the sesquiterpenoid precursor farnesyl diphosphate (FPP) by farnesyl diphosphate synthase. FPP can undergo several possible cyclization reactions that lead to more than 300 cyclic sesquiterpene skeletons [22]. The cyclizations begin with the isomerization of FPP that leads to a wide variety of mono, di or tricyclic structural elements. These backbone structures then frequently undergo secondary modifications, often by cytochromes P450 and acyl transferases to yield the huge diversity found within the chemical family of terpenoids [30]. 

**Figure 2:**
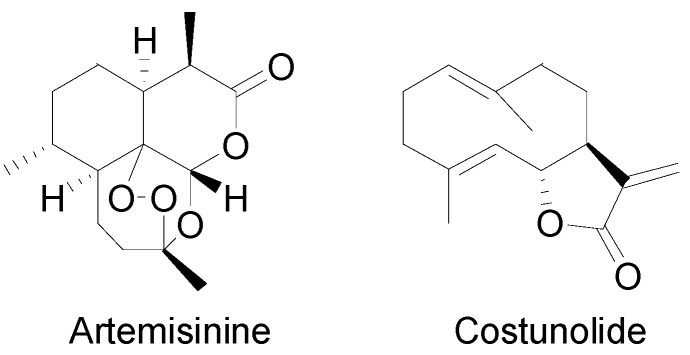
Structure of artemisinin and costunolide.

Artemisinin, produced by *Artemisia annua* L., is the best-characterized sesquiterpene lactone to date. Investigations include biosynthetic studies in microorganisms, *Nicotiana* and *Artemisia* plants [31,32,33]. Other well studied sesquiterpene lactones include costunolide from *Lactuca sativa L.* (lettuce), which has been studied in *Saccharomyces cerevisiae* and *Nicotiana* [34]. Both artemisinin and costunolide (Figure 2) were identified in the Asteraceae family and, although they are not guaianolides like thapsigargin, many of the reactions required to generate these molecules are believed to be similar to those generating thapsigargin [35]. 

### 2.2. Thapsigargin Biosynthesis

No studies have yet been able to elucidate the biosynthetic pathway of guaianolides within the Apiaceae. Even the biosynthesis of the otherwise well-studied compound thapsigargin is unknown. Given the promising results of its use as an anti-cancer drug [36], the annual demand of thapsigargin is expected to increase [37]. This encourages the development of production protocols. One possibility is the biotechnological production in a gene modified organism; an approach that requires the detailed knowledge of the biosynthesis of thapsigargin [38,39].

**Figure 3:**
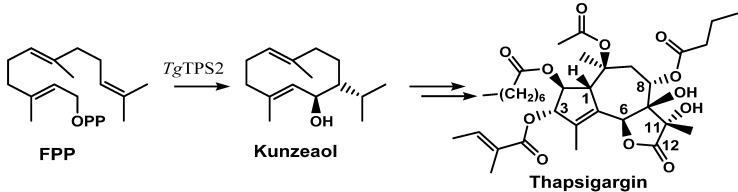
Suggested first step of the biosynthesis of thapsigargin via kunzeaol.

A terpene synthase, *Tg*TPS2, found in the transcriptome libraries (SRX096991, there is no genome sequence available) of *T. garganica* roots and fruits was found to convert FPP to kunzeaol. Kunzeaol is a probable candidate for the first biosynthetic step towards thapsigargin (Figure 3) [35]. The hydroxyl group at the C6 position of kunzeaol makes this a good substrate for the subsequent formation of the lactone ring. The following steps are thought to be similar to the ones of costunolide biosynthesis whereby the formation of the lactone ring is followed by the guaiene skeleton formation [34,40]. The enzyme family cytochromes P450 are prime candidates to participate in lactone ring formation. A triple hydroxylation on C12, as seen in costunolide, would lead to the formation of an acid group which would perform a spontaneous reaction with the OH on C6 and form a lactone ring. Cytochromes P450 might also catalyze the formation of guaiene rings by an epoxidation of the C1-10 double bond; however, this is still to be elucidated.

A number of hydroxylations are needed on positions C2, C3, C7, C8, C10 and C11, followed by the decoration of the core structure by the addition of acyl groups. Since most of the enzymes involved in this pathway are assumed to be cytochromes P450, sufficient oxygen and NADPH are crucial for the efficiency of the biosynthesis. 

## 3. Pharmacology of Thapsigargin

Thapsigargin is structurally unique. The lactone ring is a trans-anealated C7α-C6β γ-ring, which also has trans hydroxyl groups at C7 and C11 [22]. Thapsigargin, like other sesquiterpenoid lactones in general, is known for its biological activity. The main and most important pharmacological activity of thapsigargin is its potent inhibition of the SERCA pump. This effect is significant in sub-nanomolar concentrations [9,41]. The irreversible inhibition of SERCA leads to elevated cytoplasmic Ca^2+^ levels that induce apoptosis. An X-ray structure of the thapsigargin-SERCA complex provided the foundation for understanding the structural conformation of the complex, as well as the surroundings of the binding site [42]. This additionally provided detailed information for the design of a targeted prodrug with thapsigargin as the active component [42]. The prodrug is designed so that it is only activated by cancer cells, leading to apoptosis in the tumor [43].

The affinity of thapsigargin to SERCA is mainly due to lipophilic interactions between the C2 octanoyl, the C3 angeloyl-, and the C8 butanoyl-moieties and alpha-helices of SERCA. The interactions of the side-chains with SERCA have been confirmed using analogues without side-chains or inverted stereochemistry [42]. This has led to an in-depth understanding of the importance of the side-chains of thapsigargin [44]. Interestingly, when the ester group at C8 was changed from an α to a β configuration, the Ca^2+^-ATPase inhibiting properties of the molecule decreased 3000-fold [7]. Using X-ray it was shown that the C8 butanoyl group is situated in a cavity between helices when bound to the SERCA protein, a position that only allows an α configuration. It has also been shown that removing the angeloyl group or simply changing the stereochemistry of C3 leads to a significant decrease of the Ca^2+^-ATPase inhibitory effect [42]. Exchange of the acetyl group at C10 with a hydroxy moiety also decreases the biological activity of thapsigargin. Collectively, these findings show that within certain size limits, the localization of the side-chain is of more importance than its structure [16]. For the development of a prodrug, the discovery of the flexibility of the length and size of the acyl group linked to the C8-O was of great importance [42].

Since the effect of thapsigargin leads to apoptosis in any mammalian cell, thapsigargin is not suitable for use as an unmodified drug. This has been overcome by way of a prodrug strategy. The neovascular tissue of solid tumors generally, over-expresses the proteolytic enzyme Prostate Specific Membrane Antigen (PSMA). PSMA is a serine-protease that cleaves at specific amino acid motifs. Utilizing the flexibility at C8 in thapsigargin, a linker-peptide moiety that is recognized by PSMA has been attached to thapsigargin. The prodrug with the linker-peptide moiety is inactive towards the SERCA pump, but upon cleavage of the peptide moiety by PSMA the molecule becomes lipophilic and toxic. The lipophilic properties of the drug allow it to enter into the cellular membranes where it can interact with SERCA [44]. The activated drug, even with the small linker molecule, will then inhibit SERCA, which leads to apoptosis [45]. The prodrug was tested *in vivo* in nude mice, with xenografts from a human prostate cancer cell line [46]. It was observed that the level of cleaved active drug was much higher in tumor tissue than in the plasma or skeletal muscle of the mice, confirming effective drug targeting [47].

GenSpera (San Antonio, TX, USA), the company developing thapsigargin, holds several patents that cover the use of anti-cancer prodrugs that can be activated by tumors via the use of Tumor Activated Prodrug technology [48,49,50]. Initially, development was focused on treating prostate cancer by using thapsigargin coupled to a prostate specific peptide, resulting in the drug G-115 [37]. However, the design of a peptide cleaved by PSMA, which is expressed by most solid tumors expanded the use substantially and has led to the development of G-202 [43,44,51]. The difference between G-115 and G-202 is in the peptide sequences that are linked to thapsigargin. For G115 this is (starting from thapsigargin) Leu-Gln-Leu-Lys-Ser-Ser-His-Morpholine, and for G-202 it is Asp-Glu-Glu-Glu-Glu. In G-115 the PSA enzyme cleaves after the first leucine, whereas in G-202 PSMA cleaves after the first glutamic acid [43]. Focus has been shifted towards developing G-202, since this will also cover prostate cancer. Further clinical trials with G-115 are postponed, as GenSpera focuses on human trials with G-202 on patients with various types of cancer. Following the success of phase I clinical trials, the prodrug G-202 is now in phase II clinical trials for patients suffering from hepatocellular cancer (HCC) [43]. In phase I it was demonstrated that the drug was safe and well tolerated in patients with advanced stages of HCC and prolonged disease stabilization was observed [51]. G-202 or Mipsagargin is expected to be launched on the market in the coming years [37].

## 4. Production Platforms for Thapsigargin

The demand of thapsigargin is increasing given its potential medical application as a chemotherapeutic prodrug [37]. Currently, all of the commercially available thapsigargin is obtained from the fruits and roots of wild populations of *T. garganica* [52,53]. The demand for thapsigargin is expected to exceed the levels of current production, which may lead to a high harvest pressure that could endanger the species [52,54]. Therefore, one of the major challenges will be to establish new production platforms to meet the potential market demand for thapsigargin.

### 4.1. Agricultural Production

Cultivation of *Thapsia* has been shown to be complicated. *T. garganica* is difficult to germinate from seeds and to maintain under greenhouse conditions [52,54]. With successful germination, handling of the seedlings has to be done with care, since the roots are very fragile. ThapsIbiza, a Spanish company based on Ibiza has started a small production of *T. garganica* plants. Recently a method for the extraction of large quantities of thapsigargin was published enabling large scale production [55]. 

### 4.2. Organ Cultures for Thapsigargin Production

Micropropagation provides an alternative source for the production of thapsigargin [56]. Jäger *et al.* reported the establishment of *T. garganica* in *in vitro* cultures, for the purpose of producing thapsigargin [54]. Calli and suspension cultures of *T. garganica* were induced and underwent treatments with different elicitors, without successful production of thapsigargin [52]. The fact that thapsigargin is found in the resin present in specific secretory canals in the plant, may indicate the necessity of differentiation for the synthesis and storage of the bioactive compound [6]. Consequently, somatic embryos were induced and accumulated the two guaianolides, nortrilobolid and trilobolid, in the cotyledonary stage [54]. 

The formation of shoots directly from petiole and leaflet explants has also been studied [52]. A 60% rooting frequency was noted after 10 days when roots were submerged in MS liquid medium with plant growth regulator [56]. The ability to initiate prolific root formation may be advantageous [52], since roots have the ability to produce thapsigargin [57]. Hence, transgenic hairy roots are potentially an alternative source for this purpose [58]. 

### 4.3. Production in Heterologous Hosts

A variety of heterologous hosts may be suitable for large-scale production of thapsigargin or precursors thereof. *Saccharomyces cerevisiae* (yeast) is one option inspired by the efforts on the production of artemisinin. Several modifications using yeast are well described for high terpenoid production e.g., overexpression of up-stream terpenoid specific genes [32]. The green cell production system of the moss *Physcomitrella patens* has likewise been shown to be a promising producer of both sesquiterpenoids and diterpenoids [59,60], and could serve well as a production host for thapsigargin [38,39]. *P. patens* has several advantages since it can be grown in sterile cultures [39] and on a simple liquid or solid inorganic medium without phytohormones, vitamins or a carbon source [61]. Furthermore, *P. patens* performs homologous recombination with high efficiency [62], enabling the development of a stable production strain that does not require crossing steps or regeneration of whole plants [63]. The main obstacle, regardless of the choice of host organism, is the discovery of the thapsigargin biosynthetic genes.

### 4.4. Chemical Synthesis of Thapsigargin

The biological activity of thapsigargin makes it a viable drug candidate and this has led to efforts to chemically synthesize it and numerous analogues [36,45,64,65,66,67,68]. Despite the successful synthesis of thapsigargin, as well as other bioactive compounds with complex chemical structures, a commercially feasible synthetic route to these high value compounds remains a challenge. In 2004, attempts to prepare the guaianolide skeleton of thapsigargin commenced and in 2007 the first total synthesis of thapsigargin was reported [69,70]. The approach allowed for the total synthesis of thapsigargin in 42 steps from (s)-carvone with an overall yield of 0.6% [70]. The weakness of this method is the lack of a strategy for obtaining the core of the structure in a few steps and the cost of the initial starting material. 

Alternative methods include synthesizing 7,11-dihydroxyguaianolide, an intermediate in the synthesis of thapsigargin, in six steps starting from (+)-dihydrocarvone and ethyl vinyl ketone [71]. 7,11-dihydroxyguaianolide possesses five of the eight chiral centers present in thapsigargin and additionally allows for simple modifications of the C2 and C8 positions [71]. In 2012, Tap *et al.* developed the functionalized bicycledecadienone ring system of thapsigargin through a Pauson-Khand annulation reaction, however, the lactone ring and the oxygen atom on the C8 carbon atom were not incorporated [72]. 

## 5. Conclusions

Focus on the production of the upcoming drug thapsigargin will continue. The latest developments suggest that the future need will be met by plant tissue cultures along with the plants being cultivated on Ibiza. Approaches utilizing semi-synthesis or total synthesis are currently far from being economically feasible. Nonetheless, future research might provide new strategies for such approaches and open up for an extended use of thapsigargin. New prodrugs are currently being developed towards certain cancer types, and as a whole, the use of thapsigargin as a drug and chemical compound will increase in the next decade.
